# Supplementary material for: Nimodipine Used with Vincristine: Protects Schwann Cells and Neuronal Cells from Vincristine-Induced Cell Death but Increases Tumor Cell Susceptibility
Source: Int J Mol Sci. 2024 Sep 27;25(19):10389. doi: 10.3390/ijms251910389 (PMC11476576; doi:10.3390/ijms251910389)
Supplement: Supplementary file 1 [file ijms-25-10389-s001.zip › ijms-3227011-supplementary.pdf]

Supplement:

Table S1: Quantification of microscope imaging of neuronal cells. Schwann cells and cancer cell lines

	NucBlue			CellRox		
**Cell Line**	**Control**	**VIN**	**VIN + NIM**	**Control**	**VIN**	**VIN + NIM**
**RN33B**	100%	24.96%	31.16%	100%	25.17%	40.01%
**SW10**	100%	18.39%	65.91%	100%	5.49%	8.51%
**A549**	100%	38.10%	26.53%	100%	50.23%	25.17%
**SAS**	100%	41.52%	28.03%	100%	42.95%	20.74%
**LN229**	100%	55.70%	41.68%	100%	43.16%	37.26%

Table S2. Statistical evaluation of the LDH assay for the cell line RN33B

**Tukey's multiple comparisons test**	**Mean Diff.**	**95.00% CI of diff.**	**Summary**	**Adjusted P Value**
24h				
**vehicle vs. 20 µM NIM**	-0.2365	-16.41 to 15.94	ns	>0.9999
**vehicle vs. 1 µM VCR**	-19.68	-35.85 to -3.503	*	0.0147
**vehicle vs. 1 µM VCR + 20 µM NIM**	-8.578	-24.75 to 7.596	ns	0.4506
**20 µM NIM vs. 1 µM VCR**	-19.44	-35.61 to -3.266	*	0.0160
**20 µM NIM vs. 1 µM VCR + 20 µM NIM**	-8.342	-24.52 to 7.832	ns	0.4739
**1 µM VCR vs. 1 µM VCR + 20 µM NIM**	11.10	-5.076 to 27.27	ns	0.2423
48h				
**vehicle vs. 20 µM NIM**	0.1485	-16.03 to 16.32	ns	>0.9999
**vehicle vs. 1 µM VCR**	-57.95	-74.13 to -41.78	****	<0.0001
**vehicle vs. 1 µM VCR + 20 µM NIM**	-37.65	-53.82 to -21.48	****	<0.0001
**20 µM NIM vs. 1 µM VCR**	-58.10	-74.28 to -41.93	****	<0.0001
**20 µM NIM vs. 1 µM VCR + 20 µM NIM**	-37.80	-53.97 to -21.62	****	<0.0001
**1 µM VCR vs. 1 µM VCR + 20 µM NIM**	20.30	4.130 to 36.48	*	0.0118

* $p < 0.05$; **** $p < 0.0001$; ns, not significant

Table S3. Statistical evaluation of the LDH assay for the cell line SW10

**Tukey's multiple comparisons test**	**Mean Diff.**	**95.00% CI of diff.**	**Summary**	**Adjusted P Value**
24h				
**vehicle vs. 20 µM NIM**	-2.197	-10.56 to 6.162	ns	0.8745
**vehicle vs. 1 µM VCR**	-9.637	-18.00 to -1.277	*	0.0212
**vehicle vs. 1 µM VCR + 20 µM NIM**	-8.888	-17.25 to -0.5280	*	0.0352
**20 µM NIM vs. 1 µM VCR**	-7.440	-15.80 to 0.9201	ns	0.0902
**20 µM NIM vs. 1 µM VCR + 20 µM NIM**	-6.690	-15.05 to 1.669	ns	0.1421
**1 µM VCR vs. 1 µM VCR + 20 µM NIM**	0.7493	-7.610 to 9.109	ns	0.9938
48h				
**vehicle vs. 20 µM NIM**	1.286	-7.074 to 9.645	ns	0.9706
**vehicle vs. 1 µM VCR**	-17.87	-26.23 to -9.515	****	<0.0001
**vehicle vs. 1 µM VCR + 20 µM NIM**	-3.160	-11.52 to 5.199	ns	0.7053
**20 µM NIM vs. 1 µM VCR**	-19.16	-27.52 to -10.80	****	<0.0001
**20 µM NIM vs. 1 µM VCR + 20 µM NIM**	-4.446	-12.81 to 3.914	ns	0.4483
**1 µM VCR vs. 1 µM VCR + 20 µM NIM**	14.71	6.355 to 23.07	***	0.0006

* $p < 0.05$; *** $p < 0.001$; **** $p < 0.0001$; ns, not significant

Table S4. Statistical evaluation of the LDH assay for the cell line A549

Tukey's multiple comparisons test	Mean Diff.	95.00% CI of diff.	Summary	Adjusted P Value
24h				
**vehicle vs. 20 µM NIM**	1.263	-10.21 to 12.74	ns	0.9888
**vehicle vs. 1 µM VCR**	0.1741	-11.30 to 11.65	ns	>0.9999
**vehicle vs. 1 µM VCR + 20 µM NIM**	-5.529	-17.00 to 5.946	ns	0.5296
**20 µM NIM vs. 1 µM VCR**	-1.088	-12.56 to 10.39	ns	0.9927
**20 µM NIM vs. 1 µM VCR + 20 µM NIM**	-6.792	-18.27 to 4.683	ns	0.3589
**1 µM VCR vs. 1 µM VCR + 20 µM NIM**	-5.703	-17.18 to 5.771	ns	0.5045
48h				
**vehicle vs. 20 µM NIM**	-4.775	-16.25 to 6.700	ns	0.6412
**vehicle vs. 1 µM VCR**	-30.62	-42.10 to -19.15	****	<0.0001
**vehicle vs. 1 µM VCR + 20 µM NIM**	-41.45	-52.92 to -29.97	****	<0.0001
**20 µM NIM vs. 1 µM VCR**	-25.85	-37.32 to -14.37	****	<0.0001
**20 µM NIM vs. 1 µM VCR + 20 µM NIM**	-36.67	-48.15 to -25.20	****	<0.0001
**1 µM VCR vs. 1 µM VCR + 20 µM NIM**	-10.82	-22.30 to 0.6509	ns	0.0680

**** $p < 0.0001$; ns, not significant

Table S5. Statistical evaluation of the LDH assay for the cell line SAS

Tukey's multiple comparisons test	Mean Diff.	95.00% CI of diff.	Summary	Adjusted P Value
24h				
**vehicle vs. 20 µM NIM**	-1.051	-3.825 to 1.723	ns	0.7037
**vehicle vs. 1 µM VCR**	-2.437	-5.211 to 0.3371	ns	0.0957
**vehicle vs. 1 µM VCR + 20 µM NIM**	-4.752	-7.526 to -1.979	***	0.0008
**20 µM NIM vs. 1 µM VCR**	-1.385	-4.159 to 1.388	ns	0.5004
**20 µM NIM vs. 1 µM VCR + 20 µM NIM**	-3.701	-6.475 to -0.9273	**	0.0074
**1 µM VCR vs. 1 µM VCR + 20 µM NIM**	-2.316	-5.090 to 0.4582	ns	0.1196
48h				
**vehicle vs. 20 µM NIM**	-2.225	-4.999 to 0.5489	ns	0.1408
**vehicle vs. 1 µM VCR**	-4.700	-7.474 to -1.926	***	0.0009
**vehicle vs. 1 µM VCR + 20 µM NIM**	-9.132	-11.91 to -6.358	****	<0.0001
**20 µM NIM vs. 1 µM VCR**	-2.475	-5.249 to 0.2988	ns	0.0891
**20 µM NIM vs. 1 µM VCR + 20 µM NIM**	-6.907	-9.681 to -4.133	****	<0.0001
**1 µM VCR vs. 1 µM VCR + 20 µM NIM**	-4.432	-7.206 to -1.658	**	0.0016

** p < 0.01; *** p < 0.001; **** p < 0.0001; ns, not significant

Table S6. Statistical evaluation of the LDH assay for the cell line LN229

Tukey's multiple comparisons test	Mean Diff.	95.00% CI of diff.	Summary	Adjusted P Value
24h				
**vehicle vs. 20 µM NIM**	-1.243	-3.500 to 1.014	ns	0.4191
**vehicle vs. 1 µM VCR**	-10.04	-12.30 to -7.787	****	<0.0001
**vehicle vs. 1 µM VCR + 20 µM NIM**	-15.42	-17.68 to -13.16	****	<0.0001
**20 µM NIM vs. 1 µM VCR**	-8.801	-11.06 to -6.544	****	<0.0001
**20 µM NIM vs. 1 µM VCR + 20 µM NIM**	-14.18	-16.44 to -11.92	****	<0.0001
**1 µM VCR vs. 1 µM VCR + 20 µM NIM**	-5.378	-7.635 to -3.121	****	<0.0001
48h				
**vehicle vs. 20 µM NIM**	-2.330	-4.587 to -0.07318	*	0.0418
**vehicle vs. 1 µM VCR**	-46.72	-48.98 to -44.46	****	<0.0001
**vehicle vs. 1 µM VCR + 20 µM NIM**	-54.92	-57.18 to -52.67	****	<0.0001
**20 µM NIM vs. 1 µM VCR**	-44.39	-46.65 to -42.13	****	<0.0001
**20 µM NIM vs. 1 µM VCR + 20 µM NIM**	-52.59	-54.85 to -50.34	****	<0.0001
**1 µM VCR vs. 1 µM VCR + 20 µM NIM**	-8.203	-10.46 to -5.946	****	<0.0001

* $p < 0.05$; **** $p < 0.0001$; ns, not significant

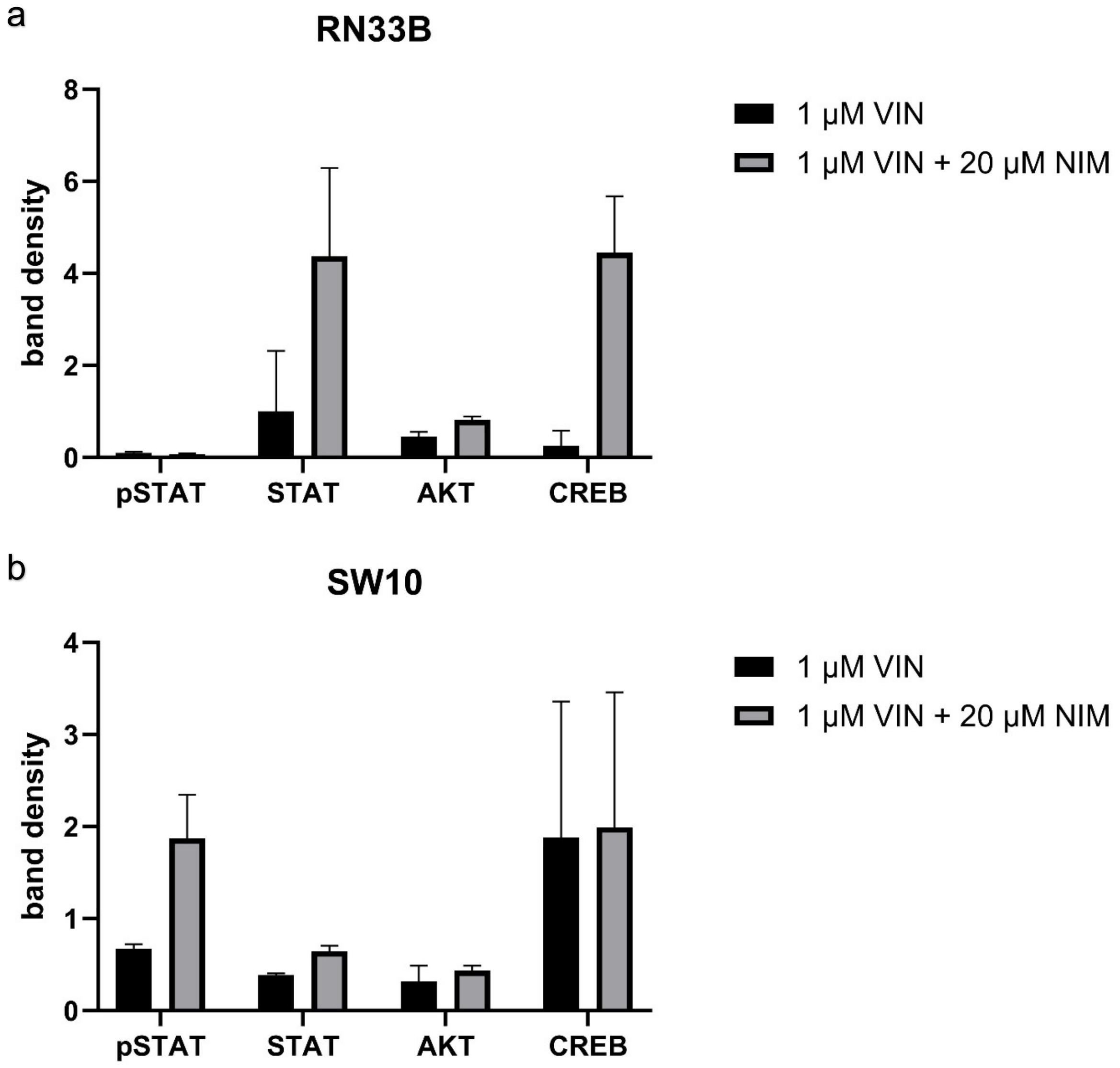

Figure S1: Quantification of pSTAT, STAT, AKT and CREB. Quantification of the bands in RN33B (a) and SW10 (b) was normalized to the GAPDH control. The mean values including standard deviation are shown. Two independent biological replicates were analyzed.

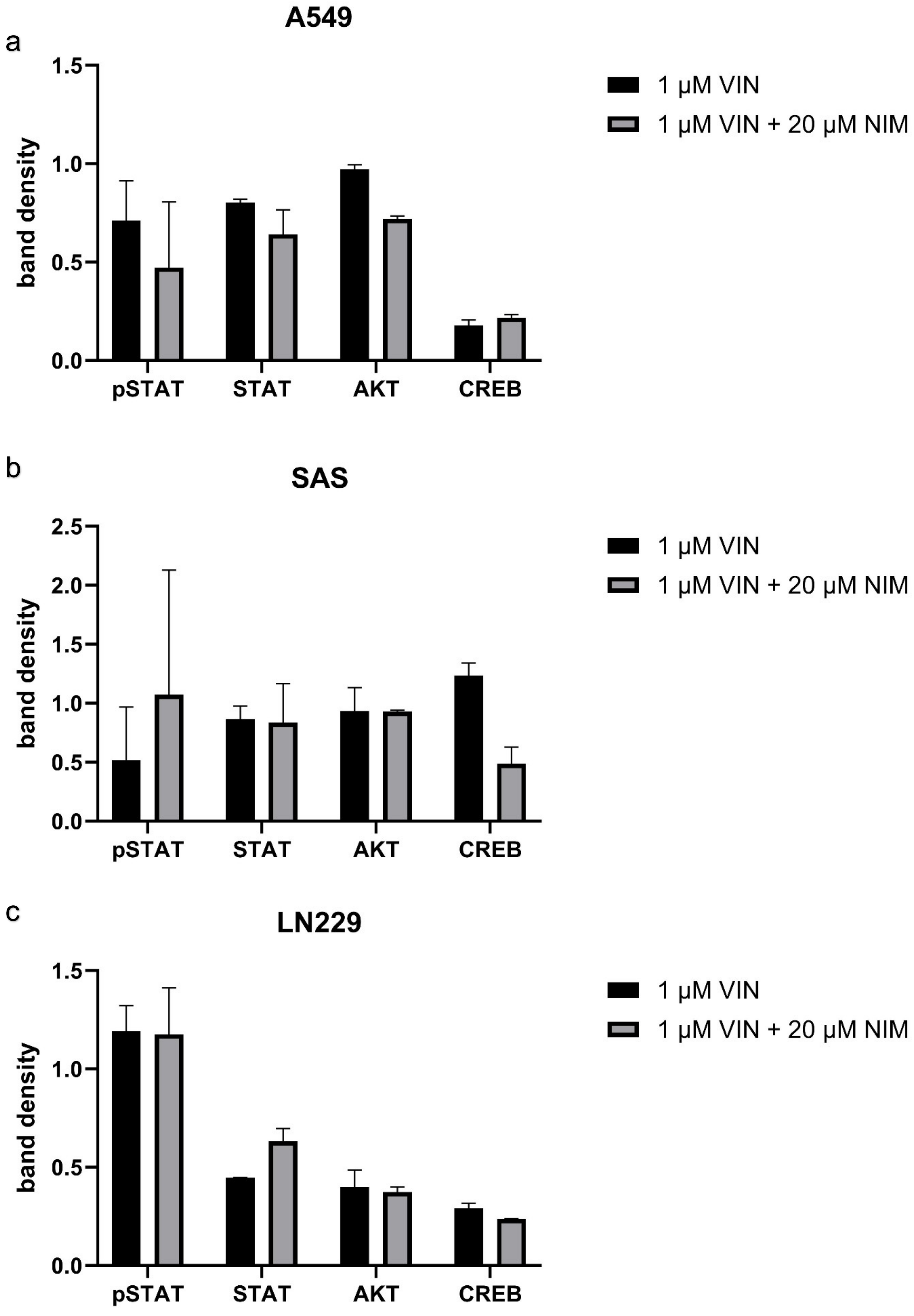

Figure S2: Quantification of pSTAT, STAT, AKT and CREB in A549 (a), SAS (b) and LN229 (c). Quantification of the bands normalized to the GAPDH control. The combination treatment was also normalized to the mon-otherapy. The mean values including deviation are shown. Two independent biological replicates were analyzed.
